# Pelvic pain & endometriosis: the development of a patient-centred e-health resource for those affected by endometriosis-associated dyspareunia

**DOI:** 10.1186/s12911-025-02907-x

**Published:** 2025-02-13

**Authors:** Gurkiran Parmar, A. Fuchsia Howard, Heather Noga, Leah Tannock, Abdul-Fatawu Abdulai, Catherine Allaire, Sarah Lett, Jessica Sutherland, Edurne Lopez de Arbina, Lone Hummelshoj, Phillipa Bridge-Cook, Paul J. Yong

**Affiliations:** 1 Department of Obstetrics & Gynecology, University of British Columbia, Vancouver, Canada; 2 School of Nursing, University of British Columbia, Vancouver, Canada; 3 Women’s Health Research Institute, British Columbia Women’s Hospital & Health Centre, Vancouver, Canada; 4 British Columbia Women’s Centre for Pelvic Pain & Endometriosis, British Columbia Women’s Hospital & Health Centre, Vancouver, Canada; 5 Endometriosis Patient Research Advisory Board, University of British Columbia, Vancouver, CA Canada; 6 Endometriosis.org, London, UK; 7 The Endometriosis Network Canada, Toronto, Canada; 8 School of Nursing, The University of British Columbia, T201 -2211 Wesbrook Mall, Vancouver, BC V6T 2B5 Canada

**Keywords:** Endometriosis-associated dyspareunia, Endometriosis, Dyspareunia, Endometriosis and painful sex, Patient-oriented e-health, E-health development, Knowledge-to-action, Technology-enabled knowledge translation

## Abstract

**Background:**

We recognized a paucity of accessible, evidence-based, empowering patient-centred resources for those with endometriosis-associated dyspareunia. Affecting more than 50% of people with endometriosis, dyspareunia can significantly impact relationships, chronic pain and the ability to have a family. We aimed to develop a patient-centred educational website for those affected by endometriosis-associated dyspareunia.

**Methods:**

To develop a functional and meaningful website for endometriosis-associated dyspareunia, we utilized a Knowledge to Action framework, supplemented with a patient-centred research design and technology-enabled knowledge translation. Our patient partners influenced the direction and scope of the project, provided critical feedback throughout the development process, and approved website revisions prior to launch. The website was developed in five phases; (1) needs assessment interviews and focus groups with key stakeholders, (2) landscape analysis of pre-existing websites, (3) development, (4) usability testing and qualitative interviews, and (5) revisions and launch.

**Results:**

Phase 1 and 2 emphasized a need for comprehensive yet plain language explanations of pain mechanisms and strategies for pain management. Rigorous consultation with key stakeholders informed the creation of the preliminary website in phase 3. Usability testing in phase 4 identified five main categories of usability problems, most of which were considered minor. Phase 4 qualitative interviews identified users’ overall impressions of the preliminary website, including that the website could help people understand their pain and describe their pain to partners and healthcare providers, as well as feel empowered to seek healthcare and validated in their experiences. User suggestions, combined with usability testing, informed revisions in phase 5.

**Conclusion:**

We developed an educational website for endometriosis-associated painful sex where people can find evidence-based etiologies for pain, pain management options, and actionable resources. Based on the data collected through qualitative interviews with patients, this website can potentially empower people to seek health care. The strength of the website development approach used was the inclusion of qualitative user insights in addition to the commonly completed user tests. The patient interviews provided insights into the potential impact of the website and, thus, ensured that we not only created a functional website that meets end users’ needs, but a website that is also meaningful to those affected by this condition.

**Supplementary Information:**

The online version contains supplementary material available at 10.1186/s12911-025-02907-x.

## Background

Endometriosis is an inflammatory disease characterized by lesions of endometrial-like tissue outside the uterus, commonly in the pelvic cavity [1]. This condition is estimated to affect 10% of reproductive age women and an unmeasured number of gender-diverse people, however, incidence varies geographically [2]. The symptoms associated with endometriosis and its severity are heterogeneous in nature, including dysmenorrhea, chronic pelvic pain, dyschezia, dysuria, fatigue, and dyspareunia [2].

Approximately half of those with endometriosis experience deep dyspareunia making this a cardinal symptom of the condition [3]. Many report a pattern of pain with sex that has resulted in unpleasant or unfulfilling sexual experiences, avoidance of sex, and reduced self-esteem, anxiety, guilt and embarrassment [4, 5]. The significant physical and emotional impacts of dyspareunia can lead to low sexual arousal, desire and satisfaction, which can further contribute to the severity of pain [6].

Despite the prevalence and burden of endometriosis-associated dyspareunia, patient-provider discussions about this specific symptom do not always occur [4, 5]. Reasons why people with endometriosis might not speak to a healthcare provider about dyspareunia include feeling embarrassed or uncomfortable discussing pain with sex as well as thinking that they cannot be helped [5]. Further, the connection between dyspareunia and endometriosis is not always made, even when the symptoms are discussed [4]. The stigma of compromised female sexual function can further prevent these discussions, and, in the absence of conversations, leaves patients ill-informed of the etiology of their pain and potential treatments.

The world wide web is an important source of public health-related information. Approximately 70% of Canadians use the web to obtain medical information [7]; behaviour that is partly motivated by the desire for greater health literacy, the availability of large amounts of information, and the convenience of locating information, especially when there is limited access to traditional information sources [8]. People are also able to anonymously find information about sensitive topics, such as sexual and mental health, in a private, safe space [9]. Additionally, individuals living in rural areas with limited access to primary or tertiary care are often able to access essential health information online [10]. As such, e-health tools are vital for people living in resource-limited settings. Access to e-health resources can increase an individual’s knowledge of their body, which can translate into healthcare-seeking behaviour [9] and active participation in health-related decision making [7]. However, the varying literacy requirements and quality of online health information has left a significant void for people [7]. Barriers to accessing relevant information likely arise from the sensitivity of sexual dysfunction and the 5 to 10 year average delay between the onset of endometriosis symptoms to surgical-pathological diagnosis [11].

Accessible, user-friendly, evidence-based sources of information about endometriosis generally, and dyspareunia more specifically, are necessary to facilitate understanding and self-management, to help people make informed health decisions, and to promote healthcare access.

Our study objective was to create a patient-centred, user-friendly, e-health resource for those affected by endometriosis-associated dyspareunia. Below, we provide a high-level overview of the development of what came to be called the *Pelvic Pain & Endometriosis* website (https://pelvicpainendo.ca/). We will report in-depth descriptions of specific website development aspects in future publications.

## Materials and methods

Our mixed-methods approach to create an e-health website drew on the Knowledge to Action (KTA) framework [12], supplemented with a patient-centred research design and technology-enabled KT (TEKT) [13], as detailed in Table 1. Useful in the development of prior e-health interventions [14], the KTA framework integrates concepts of knowledge creation and action, wherein (1) knowledge encompasses scientific evidence, contextual and experiential knowledge, (2) context is considered key for turning knowledge into action, (3) knowledge producers and knowledge users work collaboratively throughout the process, and (4) knowledge tools, products or other strategies (interventions) are developed that meet the needs of knowledge users [15, 16]. The knowledge users in this study were those with endometriosis experiencing dyspareunia.

We integrated a patient-oriented research design [17] throughout the project to emphasize patient-partnership in recognition that patients provide critical experience-based perspectives for self-managing dyspareunia that are essential for the creation of a meaningful website. For more information on the patient involvement within this project, please refer to the appendix. TEKT is the incorporation of digital technology, such as interactive websites, as the actionable tools of knowledge translation [13]. Digital technology is actionable in that it can be used as a medium to relay evidence-based information to empower patients to seek health care as well as manage their health [13].

**Table 1:** The knowledge-to-action framework for sex, pain & endometriosis

Steps from Knowledge-to-Action Framework [13]	Description
Identify problem, then identify, review, & select knowledge	Our review of research evidence pointed to: • The immense physical and psychosocial burden of endometriosis-associated dyspareunia [4, 18] consists of challenges with intimate relationships, poor quality of life, difficulties conceiving and feelings of isolation, embarrassment, and guilt [4]. • The importance of health literacy as a determinant of health for those who have complex, chronic, conditions [19], but limited opportunities or resources to support the health literacy of those with endometriosis. • Improving knowledge about endometriosis-associated dyspareunia has the potential to improve health-seeking behaviour, reduce patient distress, and improve health outcomes [20]. • No e-health resources existed to communicate sufficient quality information specific to endometriosis-associated dyspareunia.
Adapt knowledge to local context	In our Canadian context we considered that: • There is on average a 5.4-year delay from symptom onset to diagnosis and finally surgical-pathological confirmation of endometriosis [11]. Not having a diagnosis can prevent patients from accessing information pertinent to pain management. • There is a high level of internet access.
Assess barriers to knowledge use	Access to evidence-based, health related information about symptoms and management is vital for the self-management of endometriosis [21]. We were not able to locate a pre-existing health-related website that is solely focused on providing in-depth, patient-centred information about endometriosis-associated dyspareunia. There is a need to create and deliver accessible and usable information about dyspareunia to support patient self-management. Common barriers among health websites includes the lack of plain language terms and the limited reliability and usability of the presenting content [22]. We conducted a landscape review of pre-existing websites that provide a broad range of information about endometriosis to identify other barriers to knowledge use.
Select, tailor, implement interventions & monitor knowledge use	We chose to create an online health website because websites provide easily accessible health information that the public can locate and explore as needed. We used a patient-centred approach that included: • An initial focus group interview with patient partners to determine the website scope, aesthetics, content, and main messages. • An iterative process of obtaining and incorporating patient-partner feedback while creating the preliminary website. • Qualitative analysis of interviews with patients that viewed the website for the first time to determine its functionality and usability. • Qualitative analysis of interviews with patients that viewed the website for the first time to determine their perspectives of the preliminary website and identify content priorities for future development. • A final focus group review of the finished product with the patient partners for approval to launch.
Evaluate outcomes	To evaluate the effectiveness and reach of the final product, we paired the website with Google analytics, which will provide for an ongoing evaluation of key website metrics including the geographics of the audience and behaviour of users (i.e., unique visitors, pages accessed, time spent on each page, engagement rate, and links or interactive features used). Using the final product, we plan to complete a social media campaign to expand the reach of this website to a larger audience.
Sustain knowledge use	Research in endometriosis has been steadily expanding and we plan to complete yearly revisions to the website to include information from emerging research and resources.

**Fig. 1:**
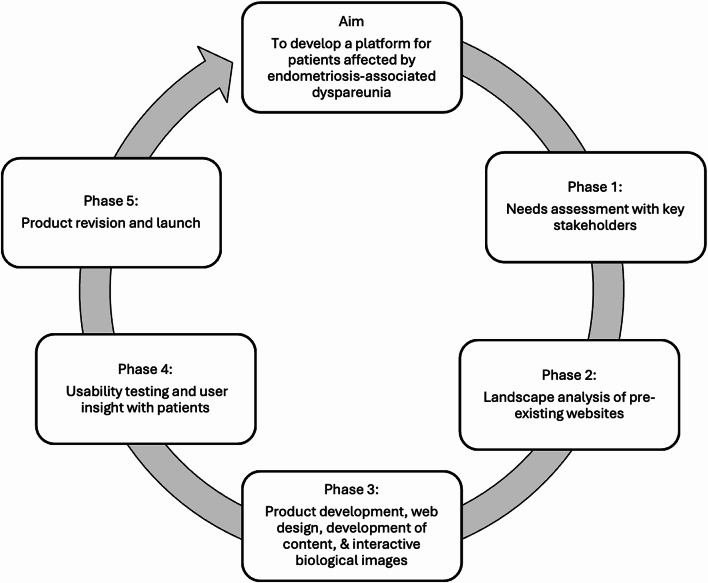
Schematic depicting the five phases of e-health resource development

We completed the development of the e-health website in five phases (Fig. 1): (1) needs assessment; (2) landscape analysis; (3) product development; (4) usability testing and qualitative interviews; (5) product revision and launch. Our collaborative team included knowledge producers, consisting of endometriosis researchers (*n* = 6) and health care providers (*n* = 6), and knowledge users, including members of a patient research advisory board (*n* = 3), and community organizations (*n* = 2). Our team also included Tactica Interactive, a digital media company.

### Phase 1: Needs assessment

We conducted in-depth, in-person focus groups or individual interviews with knowledge producers and users to identify foundational needs for developing a patient-centred website about endometriosis-associated dyspareunia. Knowledge producers included endometriosis researchers and health care providers and knowledge users included members of a patient research advisory board (PRAB) and community organizations. This included discussions of priority audiences, important topics, and preferred website ambience. Informed participant consent for this phase of the project was waived by the University of British Columbia, Children’s and Women’s Research Ethics Board.

### Phase 2: Landscape analysis

We conducted an informal review of pre-existing endometriosis-associated dyspareunia-related websites in collaboration with Tactica Interactive, a digital media company. The terms *endo*,* endometriosis*,* sex*,* sexual*,* intercourse*,* pelvic*,* vaginal*,* pain*,* painful*,* and dyspareunia*, were used in the Chrome and Safari search engines to locate relevant websites. In reviewing existing websites, we briefly assessed methods of information delivery, usability, and visual appeal, and also identified exemplars to prevent duplication of efforts.

### Phase 3: Website development

Website development included the design of the site aesthetics, building the website structure, and creating content. Informed by the priorities identified in phase 1, evidence-based information specific to endometriosis and dyspareunia was then translated into plain language by the research team and reviewed by the PRAB for complexity and healthcare providers for accuracy through informal focus group interviews. Informed consent for the PRAB reviewers in phase of the project was waived by the University of British Columbia, Children’s and Women’s Research Ethics Board.

### Phase 4 A: Usability testing

We examined how the website met users’ needs in terms of navigation, comprehensiveness of graphics and text, information content, and overall system understandability. Participants were recruited from the Endometriosis Pelvic Pain Interdisciplinary Cohort Data Registry (EPPIC) of a large urban healthcare centre in western Canada. During the time of recruitment, there were approximately 300 patients diagnosed with endometriosis who were registered in EPPIC, and who consented to be contacted for future research. We used a systematic sampling approach and selected every 11th person from a list of approximately 300 patients in the data registry. Forty-five participants were subsequently contacted.

Participants were included if they; (1) were 18 years of age or older, (2) were referred to the centre between May 1, 2019 and December 31, 2019, (3) previously consented to be contacted for future research, (4) had clinically suspected or a confirmed diagnosis of endometriosis and (5) had current/previous experiences of deep sexual pain (alone or partnered).

Through the Zoom platform, participants were asked to use the website to execute five task scenarios related to information seeking on endometriosis and dyspareunia. They were asked to verbalize their thought processes, known as think-aloud observation.

All data were recorded, transcribed verbatim and analyzed using Kushniruk and Patel’s coding scheme [23]. Using the coding scheme as a guide, some labels in the initial coding scheme that could not be labelled with any usability problems were removed, while usability problems that emerged from the data were added to the coding scheme. Approval for the usability testing was granted by the University of British Columbia, Children’s and Women’s Research Ethics Board (H19-03556).

### Phase 4B: Qualitative user insights

Patient-perspective evidence generated through qualitative research methods was considered vital to ensuring the patient-centredness of website revisions. The aim of this phase was to describe: (a) the challenges experienced by people with endometriosis-associated dyspareunia, and (b) strengths and limitations of the preliminary website. Study participants were recruited from the EPPIC data registry using a systematic sampling approach and we selected every 10th person on the list.

Participants were contacted if they: (1) were 18 years of age or older, (2) were clinically suspected or had a confirmed diagnosis of endometriosis, (3) had a history of dyspareunia, and (4) were English speaking and referred to the centre between May 1, 2019 and October 31, 2019. In-depth, semi-structured, one-hour interviews were conducted over the telephone using an interview guide (Table 2) after participants reviewed the preliminary website. We inductively analyzed the de-identified, transcribed data using the data management software NVivo™ version 12.

A research team member read the transcripts and identified broad patterns to create a preliminary coding framework. This coding framework was discussed with the study investigator, revised accordingly, and applied to all the data, whereafter constant comparative techniques were used to create categories that represented study participant feedback. Approval was granted by the University of British Columbia, Children’s and Women’s Research Ethics Board (H19-03507).

**Table 2:** Guide for semi-structured qualitative interviews

Topic	Questions
Challenges experienced by people with dyspareunia	What advice would you give to other people to help them manage or deal with challenges related to painful sex?
Strengths and limitations of the website	What are your thoughts about the website?
	Was the information new to you?
	Was the information helpful? Would it have been helpful earlier in your journey?

### Phase 5: Product revision and launch

Recommendations for revisions based on the usability testing in phase 4 A and qualitative interviews in phase 4B were presented to the research and technical design teams for consideration. Following the revisions, the second version of the website was presented to the knowledge producers and users for final review of complexity and accuracy. Prior to the launch of the website, it was registered with Google Analytics - a tracking tool to monitor the usage and ongoing activity within the website. This will give us information such as the number of current and lifetime visitors, broad location of the user’s IP address, the average time a visitor spends on the website, the sections where users are spending their time, interaction within the webpage, and more [24].

## Results

### Phase 1: Needs assessment

Three focus groups and two interviews were conducted with key stakeholders (knowledge producers and users) between January - March 2019, with the results summarized in Table 3. Knowledge producers included endometriosis researchers (*n* = 6) and health care providers (*n* = 6), and knowledge users included members of a patient research advisory board (*n* = 3), and community organizations (*n* = 2).

**Table 3:** Summary of results from the stakeholder interviews

Priority Website Audience	Important Website Topics/Content	Website Ambience
• People with and without prior endometriosis diagnosis • People with pain • People who have not seen a tertiary referral clinic • People outside large urban centres • Inclusive of sexual orientations and gender identity • People with low sexual function or low quality of life • Partners, family, and friends • Partners who feel powerless	• Types of painful sex • Causes of pain • Treatment options • Psychological impacts of painful sex • Lived experiences with painful sex • Messages related to how common painful sex is, management of pain is an individual experiences, the role partners play in supporting patients • Communication strategies • De-stigmatizing and myth dispelling messages • Actionable and proactive methods for improving health • Information for partners • Information to facilitate finding an endometriosis specific healthcare provider • Information free from overt bias	• Positive messages • Plain language which served to simplify complex medical terminology • Inclusive language • Infographics/images • Interactive features/images • Hope • Optimism • Diversity • Sense of connection • Empowerment through interactive features such as the endometriosis slider and pop-up medical terminology on medical diagrams • Validation

### Phase 2: Landscape analysis

We reviewed 17 websites that contained information about endometriosis and dyspareunia. We could not find a website that was solely dedicated to providing information about endometriosis-associated dyspareunia. Most websites had a single webpage with a brief description of the etiology and limited discussion of evidence-based treatment and self-management options for dyspareunia.

### Phase 3: Website development

The preliminary website contained eight pages as follows; (1) a home page where viewers are introduced to the website, (2) an endometriosis page where a general description of endometriosis could be found, along with an interactive diagram, (3) a painful sex page that included a detailed description of the different types of painful sex, along with interactive diagrams, (4) a mechanisms page where a thorough description of how changes in the nervous system and emotions and experiences contribute to pain with sex, (5) a treatments page that included a description of the available pain management options and links to guidelines set out by different countries, (6) a resources page that included links to global community networks and other educational websites about sex, (7) a frequently asked questions page that included answers to common questions about endometriosis, painful sex and fertility, and (8) an about us page that described the website creation team.

### Phase 4 A: Usability testing

We completed usability testing with 12 participants who all self-identified as heterosexual, were from a large metropolitan area, and ranged in age from 30 to 63 years (mean age 39 years). Participant descriptive characteristics are listed in Table 4. For this phase of the study, we did not collect data on relationship status or diagnosis because these variables were not applicable to someone’s ability to use and navigate through the website.

Our findings revealed 31 unique usability problems categorized under Kushniruk and Patel’s (2004) five main usability categories. These included problems related to; (1) navigation or finding desired information, icons, and labels; (2) participants’ understanding of labels, icons, and content; (3) timely systems’ response; (4) content, and (5) a mismatch between the users’ expectations of the type of content found through hyperlinks to different pages within the website compared to the actual content found through these hyperlinks. Except for the absence of a search bar and confusion about the word ‘mechanisms’, all other usability problems were considered minor and could be resolved through website revision to ensure a positive user experience.

Despite these minor problems, the findings suggested good overall usability and participant satisfaction with the website. These tests also revealed that this website may reduce stigma related to dyspareunia by providing emotional safety, empowering people to collaborate with healthcare providers or with other patients, validating people’s experiences and by ensuring the credibility of the content. A detailed description of the methodology, coding strategy, findings and recommendations for the usability testing have been published [25].

### Phase 4B: Qualitative user insights

We interviewed 20 people with experiences of endometriosis-associated dyspareunia, ranging in age from 18 to 44 years (see Table 4).

**Table 4:** Descriptive characteristics of the participants recruited in phase 4 A and B

Descriptive Indicator	Usability Tests	User Insights
N (percentages)	12 (100.0)	20 (100.0)
Mean Age (min, max)	38.75 (30.2, 47.3)	31.5 (18, 44)
Sexual Orientation		
Heterosexual	12 (100.0)	16 (80.0)
Bisexual	0 (0.0)	2 (10.0)
Pansexual	0 (0.0)	1 (5.0)
Homosexual	0 (0.0)	1 (5.0)
Relationship Status		
Partnered	-	17 (85.0)
Unpartnered	-	3 (15.0)
Ethnicity		
Caucasian	6 (50.0)	18 (90.0)
South Asian	0 (0.0)	1 (5.0)
South American	2 (16.7)	1 (5.0)
Indigenous	1 (8.3)	0 (0.0)
Did not disclose	3 (25.0)	0 (0.0)
Educational Level		
College	-	9 (45.0)
Graduate School	-	6 (30.0)
High School	-	4 (20.0)
Vocational School	-	1 (5.0)
Endometriosis clinically suspected or confirmed diagnosis	12 (100.0)	20 (100.0)
Previous histological diagnosis at prior surgery	-	10 (50.0)
Visual diagnosis at prior surgery	-	5 (25.0)
Current endometrioma on imaging	-	1 (5.0)
Clinically Suspected based on history and examination	-	4 (20.0)

**Fig. 2:**
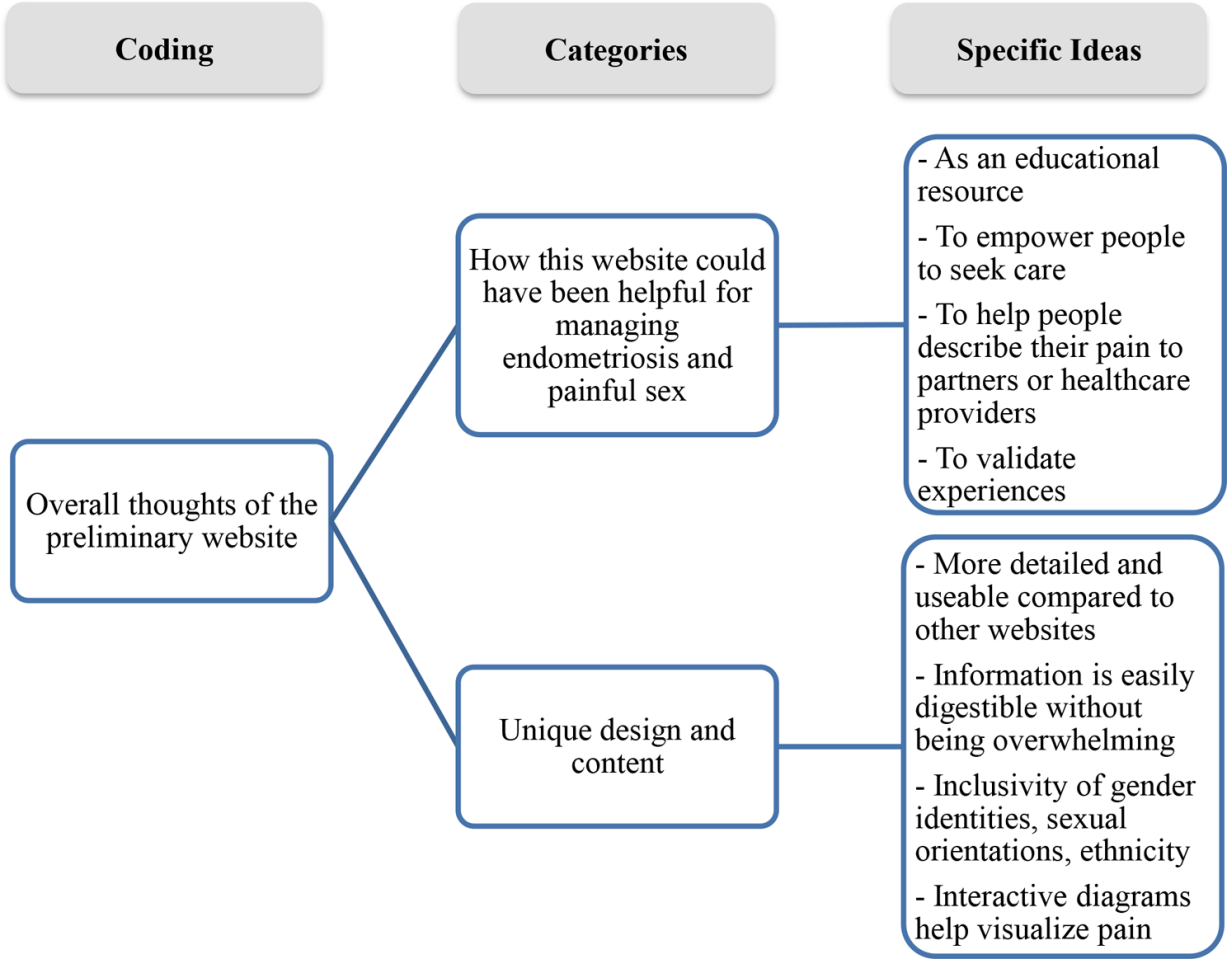
Summary of the results from the phase 4b qualitative user insights study

We categorized participant perspectives according to their overall impressions and suggestions for improving the website as depicted in Fig. 2. Overall impressions centred on the utility of the website, unique aspects of the design and content. More specifically, the participants highlighted the utility of the preliminary website as an educational resource, an important feature considering participants’ self-described limited understanding of painful sex as a symptom of endometriosis.

*“And to have a website like what you guys had like*,* had I had that*,* I think I would have been in much better shape and I’m not just saying that*,* I went through that website for 45 minutes and was like*,* wow*,* it’s answered most of my questions I had when I was not knowing what was going on.” (Participant 14)*.

According to participants, they would have used this website to help describe their pain to healthcare providers and their intimate partners if it existed when they were younger. Moreover, if the participants had had access to this website previously, they would have felt empowered to seek healthcare sooner. They further highlighted how the website validated their experiences of pain and could help others with endometriosis feel less alone.

*“I was trying to read it through a lens of like I was 17 and I found this website. Would this be something that would intrigue me or would make me feel better? I think for sure it would have been like*,* there’s a whole website on this. I’m not by myself. There’s other people!” (Participant 13)*.

Unique aspects of the website design and content that participants appreciated included the use of plain language and the pacing and spreading out of digestible rather than text-heavy, overwhelming information. This aided the comprehension and conceptualization of information. The participants also appreciated that the website was inclusive of different gender identities, sexual orientations, and ethnicities, as well as the use of interactive features and biological images to help visualize endometriosis and factors contributing to pain.

*“I know general anatomy and physiology and it’s hard to kind of know exactly where I’m feeling certain things*,* so I definitely found that [diagram] helpful.”(Participant 2)*.

The participants also provided suggestions for improving both the website content and design. Specific to content, further information about endometriosis, including a list of associated symptoms, was recommended, as were additional plain language descriptions of terms such as ‘deep penetration’ and ‘mechanisms’. There was also a suggestion to convey how information in one part of the website relates to information in another section, for example, including an explanation of how pain management options work together to reduce pain rather than presenting them as separate entities.

*“I like your four boxes…but I feel like people might be trying to visualize a holistic approach to endometriosis or a synchronized approach…. These [treatment options] come together and they synchronize to help you when you’re dealing with painful sex… These aren’t four separate tabs to be considered. They actually work together…When I look at them as boxes*,* they kind of feel separate.” (Participant 14)*.

To improve the website design, the participants recommended adding a search bar to increase the efficiency of finding specific information. They also noted that some images did not match the seriousness of the topic and suggested alternative imaging to ensure a better match with the content and sentiment of the website as well as ensuring there were images that represented a diversity of intimate relationships.

*“Going to the endometriosis page and seeing somebody’s head lovingly on their mother’s shoulder*,* that wouldn’t be comforting for me.” (Participant 11)*.

Participants suggested including more information about actionable self-management strategies or lifestyle changes such as diet and exercise, sexual positions that may reduce pain, as well as a plain language version of scientific papers, infographics, visual abstracts or educational videos they could share with others.

*“There’s a lot of things you can’t access unless you have*,* like you’re behind a firewall*,* unless you’ve got a PubMed login or something. And so if there’s public access*,* research or findings that could be shared on the website that it removes a barrier of having to figure out how to get that information otherwise.” (Participant 5)*.

*“I think infographics are great. I think that that’s a great way to try to put things visually for people. I think it’s important for people who don’t understand how to read research”. (Participant 11*)

User insights that fell within the capacity of the current site development were incorporated during the Phase 5 product revisions prior to website launch.

### Phase 5: Product revisions and launch

Improvements were made in each of the five categories identified by the usability testing. (1) Navigation problems, including broken or missing links, were fixed and the clickable features were improved. A search bar was added to the website for ease of finding desired information. (2) The icons, labels and content that respondents found challenging were improved by translating terminology into plain language and by including more detailed information. For example, the term ‘mechanisms’ was changed to ‘what causes painful sex.’ (3) The system’s response, the loading time of the website, was improved by Tactica. (4) Content was reorganized, and additional web pages were created to allow for more thorough plain language descriptions. (5) The sections of the text that hyperlinked to different pages of the website and the corresponding redirections were altered to better match the expectations of the participants. Some examples of the revisions made are included in Table 5. Additionally, a search bar and links for sharing content on social media were added to each page.

**Table 5:**
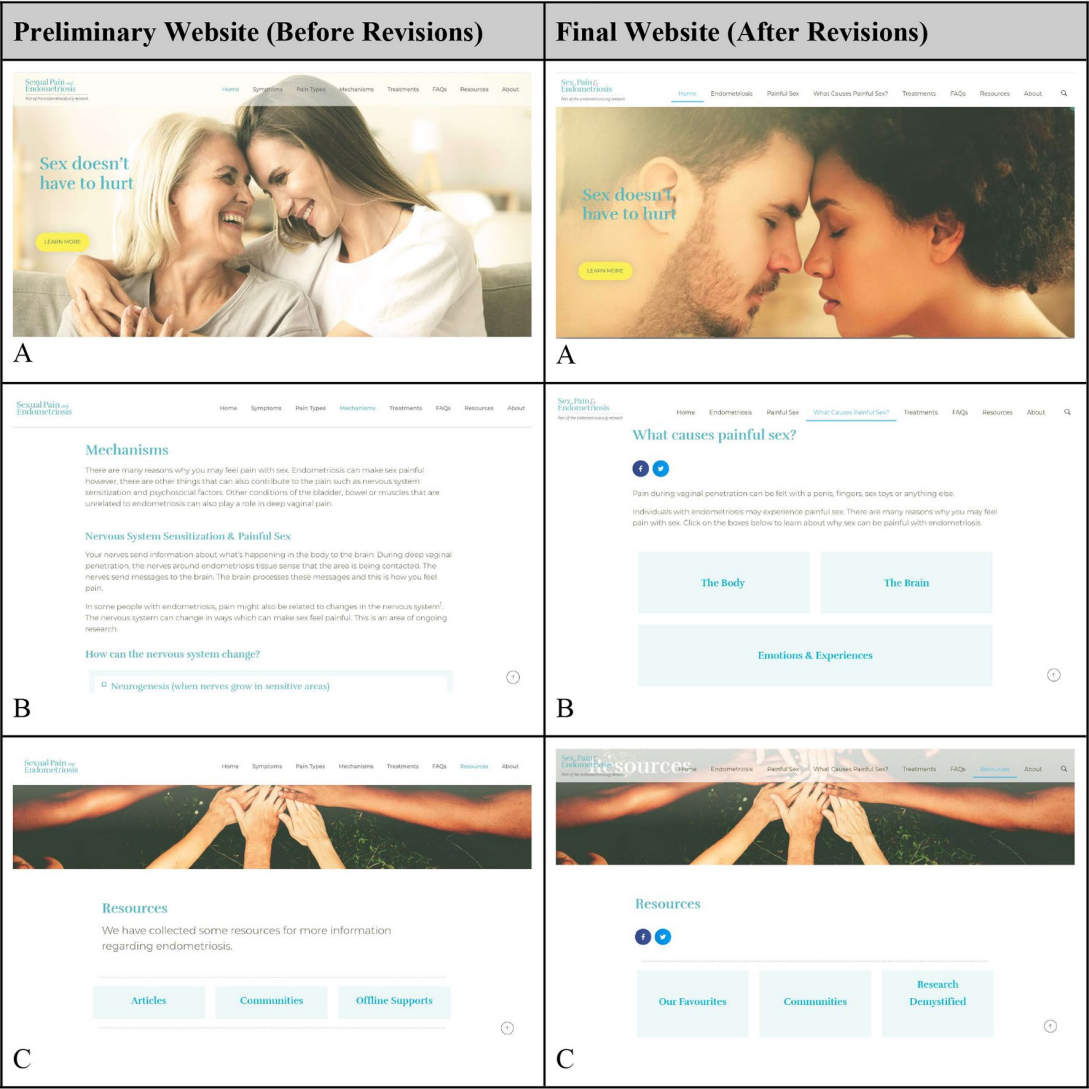
Images of the website before and after incorporation of data from phase 4A and B

Based on the qualitative user insights, a list of other endometriosis symptoms were added to the ‘endometriosis’ page, a plain language definition of deep penetration was added, more thorough descriptions were added to the ‘treatments’ page, stock images were adjusted to match the sentiment of the condition, and an additional space was created to add visual abstracts to provide users with downloadable content on emerging research and data. We further included video explanations about endometriosis, painful sex, and nervous system sensitization created by a research trainee and a patient partner as an auditory accompaniment to the written information.

Panel A shows an example of a stock image on the main page of the website that was changed to better match the content and sentiment of the website. This panel also shows the addition of a search bar at the top right side of the image. Panel B shows an example of how terminology was changed to become more plain language. The ‘mechanisms’ tab became the ‘what causes painful sex?’ tab. Within this tab, additional sub-pages were created to highlight the different mechanisms of endometriosis-associated painful sex and to reduce information overload for the user. Panel C shows how the section for ‘articles’ on the ‘resources’ page was changed to ‘research demystified’ to create space for infographics/visual abstracts.

## Discussion

We have described the development of an educational, patient-centred, website about endometriosis-associated dyspareunia, wherein theory provided useful direction and highlighted important considerations. The knowledge-to-action framework was useful in guiding our process of conceptualizing the problem, tailoring our approach to our context, developing an e-health intervention tailored to knowledge users, and considering future monitoring, evaluation, and sustainability. Our patient-oriented research approach (Appendix Table 6), wherein endometriosis patients were involved in all phases of the process, ensured this project addressed issues of importance to patients themselves. The insights of healthcare providers, advocacy groups and lived experiences of patients were invaluable in creating a website that matched the end users’ needs. As this was a patient-oriented project, challenges faced during the design process when encountering conflicting feedback, the feedback from patients was prioritised.

We found that the user testing and insights were critical phases of the development process. User testing identified categories that required improvement in terms of functionality and information delivery on the website. User testing to assess usability and functionality of preliminary websites are commonly completed in a user-centred design approach [14, 26–29]. However, seldom do development teams complement these findings with qualitative user insights. We found the addition of interviews beneficial in identifying aspects of the website that people found useful and appreciated as unique. It allowed us to explore, on a deeper level, what users found to be meaningful. These included, the use of plain language for ease of reading, the inclusivity of diverse gender identities and sexual orientations, and the use of interactive biological images that helped people visualize their pain. Knowledge of the useful and unique aspects of the website signaled that our project processes were generating a patient-centred product, identified website aspects that should not be changed and could even be bolstered further, and enabled our team to forge ahead with essential revisions as per end users. We recognize, however, that as a result of the homogeneity of patient characteristics within the user insights, our findings may not be generalizable. Subsequent e-health development projects might consider this complementary qualitative approach along with recruitment strategies that maximize diversity. Likewise, future iterations of the website could seek to incorporate the vision of diverse communities such as those from East, South and Southeast or Indigenous backgrounds. Subsequent research may also consider generating this content through an arts-based approach to further illuminate the experiences of diverse populations with endometriosis.

There is a need for people with endometriosis to have access to health-related information [30]. While our goal was to develop an educational website, our participants highlighted the power of knowledge to inform their management of endometriosis associated dyspareunia. According to participants, this website has the potential for helping people understand dyspareunia as a symptom of endometriosis, seek healthcare, and describe their pain to their partners and healthcare providers. These findings are important considering that the link between dyspareunia and endometriosis is not always made [4], not all healthcare providers ask about this symptom [4], and there is frequently a considerable delay in time to diagnosis [11]. Moreover, participants described feeling that their experiences were validated after reading the website and they reflected that if viewed earlier in their illness trajectory, it could have helped them feel less alone. In the context of the stigma around sexual health-related conditions, and the mental health challenges among people with endometriosis [31], the de-stigmatizing and therapeutic potential of a website such as ours deserves subsequent investigation [32]. Furthermore, the link between the provision of health information and self-management has been previously described [34]. Health information has the potential to reduce anxiety, improve confidence and promote patient activation [33]. Our findings highlight the value participants saw in the information found on the website that might bolster the self-management of their endometriosis symptoms.

Despite the strengths of our approach, consistent with other e-health projects, we found that developing an e-health application is time- and resource-intensive as it is an iterative process that requires constant feedback, revisions and input from knowledge producers and users [28]. The oversight to not collect information on educational level in the user testing is also a limitation and should be considered for future projects. Further, designing anatomically accurate biological images with web developers was a challenge due to the difficulty of visualizing a complex medical condition. This rigorous process also required frequent communication between the designer, the researchers and healthcare experts to produce clear, easily digestible but accurate interactive content. While we were able to complete the majority of revisions identified through user testing and insights, there were still outstanding additions that we were unable to complete due to budget constraints. We prioritized what stakeholders considered to be essential practical information over interactive features to remain within budget. We also considered what aspects could be put on hold for future iterations (e.g. partner resources) as we continue to grow. We also found pre-allocating a sufficient budget for the phase 5 revisions to be essential for this project.

## Conclusion

Using a systematic development process outlined by the knowledge-to-action framework [12], we created the e-health website *Sex*,* Pain & Endometriosis*. This patient-centred website is inclusive of audiences diverse in age, ethnicity, gender identity, relationship status, sexual orientation, and endometriosis diagnosis.

This website contains the biological and psychological etiologies of endometriosis-associated dyspareunia as well as pain management options written in plain language, with interactive images, video explanations, and actionable resources.

### Practical implications

Developing a patient-centred website specific to endometriosis-associated dyspareunia has the potential to address many end users’ unmet needs regarding health literacy, validation for their experiences and empowerment. Users can compare their experiences with the information found on this website in private. This website can also be used as a tool for partners of patients to learn about dyspareunia. Since this website is available publicly online, it can be especially useful for people living in remote areas with limited access to endometriosis-specific healthcare providers. The main message of this website is that dyspareunia is a real symptom of endometriosis, support is available, and those affected should connect with a healthcare provider.

## Electronic supplementary material

Below is the link to the electronic supplementary material.

Supplementary Material 1

## Data Availability

The datasets used and analysed within this project are not publicly available due to restrictions from the ethics board however, they may be made available from the corresponding author for a reasonable request.
