# Supplementary material for: Pelvic pain & endometriosis: the development of a patient-centred e-health resource for those affected by endometriosis-associated dyspareunia
Source: BMC Med Inform Decis Mak. 2025 Feb 13;25:79. doi: 10.1186/s12911-025-02907-x (PMC11827241; doi:10.1186/s12911-025-02907-x)
Supplement: Supplementary file 1 — Supplementary Material 1 [file 12911_2025_2907_MOESM1_ESM.docx]

**Supplementary File**

The GRIPP2 short form is an international checklist to improve the clarity, transparency and practice of reporting patient involvement in health research [33]. The details of the GRIPP2 short form are organized in table 6.

**Table 6.** GRIPP2 short form

| **Section and Topic** | **Item** | **Reported on page No** |
| --- | --- | --- |
| 1: Aim | Report the aim of PPI in the study | 5 |
| 2: Methods | Provide a clear description of the methods used for PPI in the study | 7-9 |
| 3: Study results | Outcomes—Report the results of PPI in the study, including both positive and negative outcomes | 9-15 |
| 4: Discussion and conclusions | Outcomes—Comment on the extent to which PPI influenced the study overall. Describe positive and negative effects | 7-8 & 16-17 |
| 5: Reflections/critical perspective | Comment critically on the study, reflecting on the things that went well and those that did not, so others can learn from this experience | 16-17 |

PPI=patient and public involvement
